# The pharmacokinetic advantage of 5-methyltetrahydrofolate for minimization of the risk for birth defects

**DOI:** 10.1038/s41598-018-22191-2

**Published:** 2018-03-06

**Authors:** Steven W. Bailey, June E. Ayling

**Affiliations:** Department of Pharmacology, University of South Alabama, Rm 3370 MSB, Mobile, AL 36688 USA

## Abstract

Despite efforts to increase folic acid (FA) intake, even within countries mandating FA fortification, there remain pregnant women with folate levels inadequate to minimize congenital disorders (e.g., of the neural tube, heart, and lip/palate). The pharmacokinetics of FA and [6S]-5-methyltetrahydrofolate (5-MTHF) were examined to find a reliable and minimal dose for rapidly rescuing folate status prior to critical periods of embryonic development. Serum total folate increased much more rapidly over the first four days in insufficient women given 7.5 mg doses of 5-MTHF than the same regimen of FA (P for trend <0.0001). Nearly all women given 7.5 mg 5-MTHF (every 12 hours, five doses total) almost immediately reached 50 nM serum total folate. Moreover, this level could be maintained by subsequent administration of 0.4 mg/d of folic acid. Thus, 5-MTHF enables repletion of folate stores more quickly and uniformly than FA and without exposure to unmetabolized FA.

## Introduction

**Figure 1:**
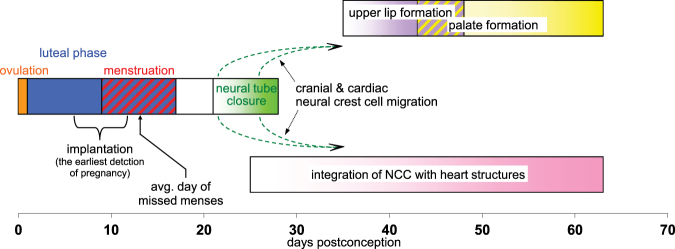
Timeline of the folate sensitive development of the human embryo. The closure of the neural tube begins about day 21 after conception with the successive fusion at several sites of the opposing tops of the now elevated neural fold. This is, on average, seven to eight days after women miss a menstrual cycle. Moreover, those using a high sensitivity pregnancy test (which detects the human chorionic gonadotropin triggered by implantation) can discover their pregnancy four to five days earlier still. The migration of the cranial and cardiac portion of neural crest cells (NCC) begins between 23–27 days after conception as they separate from the closing neural tube. Cardiac NCC contribute to much of the outflow tracts of the heart, and generate the septum between the pulmonary artery and the aorta. Other cephalic NCC travel to the head to participate in lip and palate formation. A cleft of either of these is the result of a failure of their respective right and left elements to join within the first 9 weeks. As with some heart defects clefts have been attributed, in part, to inadequate neural crest cell migration.

Low maternal folate status has been linked to the risk for several birth defects. The neural folds start to elevate 19 days after conception, and fusion of their tops begins between about 21 to 22 days (Fig. 1). If not complete by day 28, this results in neural tube defects (NTD) such as spina bifida, anencephaly, and encephalocele. Moreover, several studies suggest the risk for congenital anomalies of other structures requiring integration of migrating neural crest cells, such as the heart and the lip/palate, can also be decreased by maternal FA supplementation^1–5^. After about eight weeks cardiac outflow tracts and septation that depend on neural crest cells are complete. The lip and the palate normally develop over a six to ten week period after conception, with a cleft typically due to a developmental failure within the first nine weeks. Each of the above defects is a considerable burden on children, their parents, and society.

Early clinical trials indicated that periconceptual supplementation with folic acid (FA) considerably reduces the risk of NTD. The Medical Research Council Vitamin Study Group demonstrated that treatment with 4 mg/d FA prevented 72% of recurrent NTD compared to the control group^6^. It is now understood that the percentage of risk reduction depends, in part, on the baseline incidence within a given study population. Due to poor compliance with recommended supplementation and the slow rate which supplementation raises folate status, many countries have initiated mandatory food fortification with FA. This has been effective in decreasing the incidence of NTD. However, several studies indicate that folate fortification does not reach all women^7–10^. Moreover, folate levels are still low in countries lacking mandatory fortification. Programs encouraging pre-conceptional consumption of folic acid are typically followed by fewer than 30 percent of women. Therefore, many are still at elevated risk for a pregnancy adversely affected by folate deficiency^11^.

When pregnancy is detected, currently the only option considered for those not previously affected by a NTD birth is administration of a standard prenatal folate supplement (0.4 mg/d folic acid). But, some women are at special risk beyond that due to low folate status, for example, those who have a family history of NTD, obesity, consanguineous parents, maternal insulin-dependent diabetes, history of still births, high body temperature due to fever or hot baths early in pregnancy, taking antiseizure medications or antifolate chemotherapy agents, use of diuretics, periconceptual diarrhea. Several genetic polymorphisms, for example of 5,10-methylenetetrahydrofolate reductase, are also thought to contribute to risk in some populations. The risk for heart defects is influenced by maternal viral infection, and alcohol or cocaine use during pregnancy. A method is especially needed for decreasing birth defects for such women if folate insuffficient after conceiving. Evidence for a benefit of post-conceptional FA has been reported in studies in China and Hungary^2,12^. This is consistent with observations that embryos with incipient defects have not been observed earlier than Carnegie Stage 11^13^, suggesting that NTD results from an aberrant process that occurs during closure itself (between 21 and 28 days).

In elevating the folate of a pregnant woman, the most relevant measure of folate status must be considered. The study by Daly, *et al*. showed that risk in an Irish population was minimized for those who had red cell total folate levels greater than 906 nM (measured by microbiological assay)^14^. However, red cells do not exchange folate with other tissues. The authors of this often cited work state that “…it is clear that the fetus accesses folate through maternal plasma folate…”. Since their samples were taken about 15 weeks into pregnancy, they chose to focus on red cell folate to better reflect their subject’s preconceptional status. However, for the developing embryo, plasma folate is the relevant factor, regardless of red cell folate (which can lag behind changes in intake due to its slow turnover). Several studies have demonstrated that the recommended two months of administration of 400 μg/day FA to women of child bearing age results in 44 to 55 nM total plasma or serum folate^15–17^. Administration of 5 mg/d of FA to pregnant women has been proposed^18^, but no studies appear to have examined if this increases serum folate levels in less than two weeks. Therefore, one goal of the current study was to find a regimen of FA or 5-methyl-6S-tetrahydrofolate (5-MTHF) administration that could quickly (within days) elevate plasma folate to 50 nM. In addition, we sought to find the minimum total dose of folate that could load tissue stores to the point where the mother could be soon transferred to a standard prenatal folate supplement (e.g., 0.4 mg/d folic acid) to maintain the newly acquired plasma folate level.

## Results

### Characteristics of the study population

**Figure 2:**
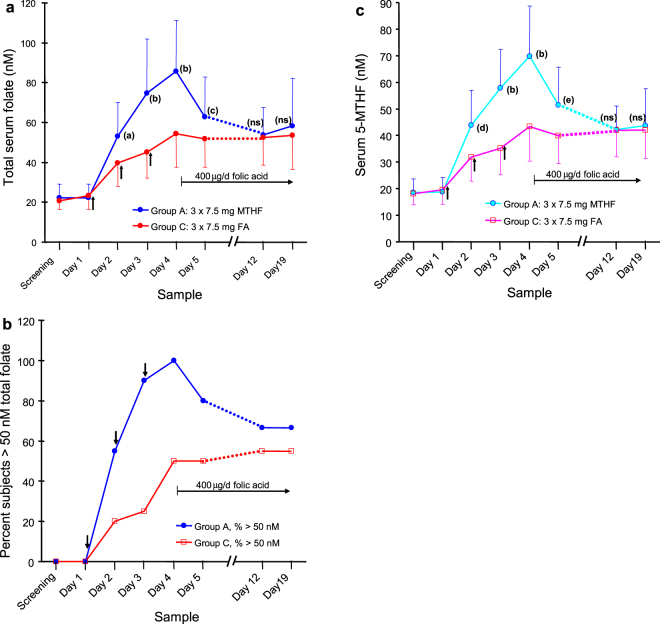
Comparison of the rate of elevation of serum folate in women administered folic acid or 5-methyltetrahydrofolate (5-MTHF). The effect in women of child bearing age of three doses of 7.5 mg of 5-MTHF or folic acid (FA) given every 24 hours (arrows) on (**a**) average serum total folate, (**b**) the percent of subjects ≥ the target serum folate of 50 nM, (**c**) average serum 5-MTHF. Subsequent to day 4, all subjects were administered 0.4 mg/d of FA. Error bars show the standard deviations. (a) P = 0.03, (b) P > 0.0005, (c) P = 0.028, (d) P = 0.025, (e) P = 0.007 (corrected for multiple comparisons).

**Figure 3:**
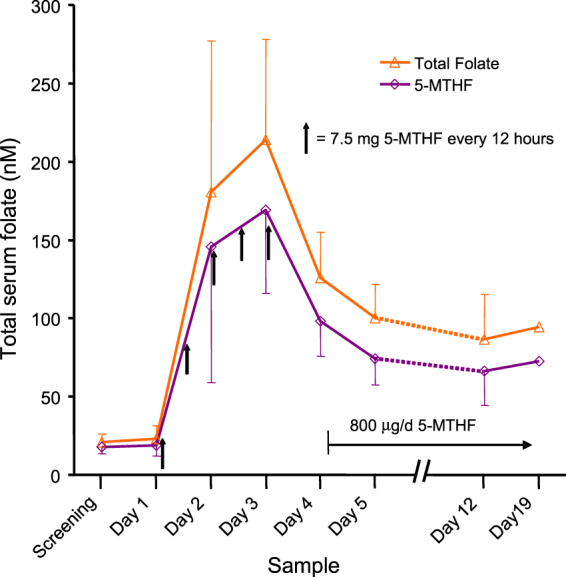
The effect in women of child bearing age of five doses of 7.5 mg of 5-MTHF given every 12 hours on the average serum total folate and serum 5-MTHF. Subsequent to day 4, all subjects were administered 0.8 mg/d of 5-MTHF. Error bars show the standard deviations.

Subjects ranged between 19 to 45 years of age, average: 28.1 ± 7 years. Self identified race was 50% white, 39% black, 1.6% Asian, 1.6% American Indian, and 8% mixed. The average total serum folate for all subjects on entry was 19.7 nM as measured by the Immulyte 2000 assay. When these samples were re-analyzed, the average total serum folate was 21.3 nM (S.D. ± 5.2) by the microbiological assay, and serum 5-MTHF was 18.1 nM (S.D. ± 4.2) by HPLC (85% of total folate). The three groups (A, B, and C) were not different by ANOVA (P = 0.81 and 0.87 for total and 5-MTHF, respectively) (Fig. 2a,b and Fig. 3 screening samples). Comparison of the screening samples for the subjects in all three groups together with the sample taken immediately before the first dose showed that serum total folate increased by 6% to 22.7 nM, and serum 5-MTHF increased 4% to 19.0 nM, perhaps due to regression to the mean, but neither increase was statistically significant (P = 0.094 and 0.21, respectively) (Fig. 2a,b and Fig. 3: day 1 samples). The standard deviations of the change between the screening sample and the first study sample were 6.5 nM for total folate and 5.0 nM for 5-MTHF.

### Elevation of serum folate and repletion of body stores with three doses of 5-MTHF

In group A serum total folate and 5-MTHF continuously increased in all subjects for each sample following each of the three doses of 7.5 mg 5-MTHF administered once each morning (Fig. 2a). Twenty four hours after the first dose 45% of subjects still had serum total folate concentrations below 50 nM, though all were above 30 nM. Twenty fours hours after the second dose serum total folate was greater than 44 nM for all subjects, with only 10% below 50 nM. A day after the third and last dose the total folate and 5-MTHF averaged 85.8 nM (S.D. ± 25.3) and 69.3 nM (S.D. ± 19.4), respectively, and the subject with the lowest response had values of 56.8 nM and 46.5 nM, respectively. However, by the next day (having been administered only 0.4 mg FA) the average serum folate declined to 62.8 nM and 51.4 nM for total folate and 5-MTHF, respectively. At this point 20% of subjects were below 50 nM serum total folate, with the lowest value being 34 nM (Fig. 2b). After a week of administration of 0.4 mg/d FA, serum total folate and 5-MTHF had fallen slightly further (53.8 nM and 42.2 nM, respectively), but were maintained at about this level over yet one more week. Folate levels in the day 12 and day 19 samples were not statistically different by paired t-test (P > 0.5). During these two weeks about a third of subjects were found to have serum total folate less than 50 nM (Fig. 2c), although only one subject was observed to be less than 35 nM. Although changes in serum 5-MTHF approximately paralleled those of total folate, the ratio of 5-MTHF to total folate fell from 0.83 before any administration to 0.76 (P = 0.011 by paired t-test) by day 19.

### The effect of five doses of 5-MTHF twice a day

In group B (subjects administered 7.5 mg of 5-MTHF every 12 hours) serum total folate and 5-MTHF increased up to an average of 213.8 nM (S.D. ± 64.2) and 168.9 nM (S.D. ± 53.1), respectively, by 12 hours after the fifth and last dose (Fig. 3). Of the samples taken during the three mornings following these doses of 5-MTHF, only a single subject (and only on the first morning) was below 50 nM serum total folate. Twenty four hours after the last dose of 7.5 mg 5-MTHF, serum total folate and 5-MTHF fell to an average of 126 nM (S.D. ± 29.3) and 98 nM (S.D. ± 22.3), respectively, but no subject was ever below 50 nM total folate. While samples taken 24 hours after a 7.5 mg dose of folate (groups A and C) will largely reflect a new homeostasis, those taken only after 12 hours also are influenced by incomplete clearance^19^. Importantly, in the two following weeks with administration of 0.8 mg per day of 5-MTHF although serum total folate and 5-MTHF decreased further to an average of about 90 nM and 69 nM, respectively, only a single subject was seen to have a total serum folate value (39 nM in the day 19 sample) of less than 50 nM. Total folate in the day 12 and day 19 samples were not significantly different (P = 0.2 by paired t-test). Changes in serum 5-MTHF approximately paralleled those of total folate, but the ratio of 5-MTHF to total folate decreased from 0.85 before any administration to 0.77 (P = 0.00021 by paired t-test) by day 19 (Fig. 3).

### The slow increase of serum folate with three doses of folic acid

Subject group C, which was treated identically to group A, except for administration of 7.5 mg of FA once a day for three days, responded much more slowly over the first four days (Fig. 2) than group A who had been given 5-MTHF (P < 0.0001 for the trend). On the morning 24 hours after the first dose, 80% of subjects had serum total folate less than 50 nM (average 39.7 nM, S.D. ± 11.6 nM) (Fig. 2c), with 35% below 35 nM. The samples on the morning after the second dose, although now elevated further to an average serum total folate of 45.2 nM, S.D. ± 12.9 nM, still showed 75% of subjects with less than 50 nM and 25% with less than 35 nM (Fig. 2b). The samples taken 24 hours after the third and final dose of 7.5 mg of FA gave the highest serum total folate and 5-MTHF of 54.4 nM (S.D. ± 16.8) and 43.4 nM (S.D. ± 13.0), respectively. However, 50% of the subjects were still below 50 nM and 10% were below 35 nM. Over the next two weeks of administration of 0.4 mg/d of FA, serum total folate and 5-MTHF did not change significantly by paired t-test (P > 0.6), 45% of subjects remained below 50 nM, and 15% were below 35 nM. The ratio of 5-MTHF to total folate decreased from 0.86 before administration of folic acid to 0.83 by day 19, but this was not significant (P = 0.63 by paired t-test).

## Discussion

Comparison of 5-MTHF with FA (7.5 mg/d for three days, groups A vs C) shows that serum total folate is 23% to 55% higher with the former over each of the four mornings after initiation of administration. In particular, 5-MTHF was more uniform in quickly increasing total folate to the putative target level. For example, 48 hours after the first dose of 5-MTHF, but just before the third dose, all 20 participants had serum total folate values ≥45 nM, and 90% were above target. On the other hand, at this same time 55% of those administered folic were still below 45 nM. After 12 days with continued administration of 0.4 mg/d of FA to both groups, serum total folate levels became indistinguishable between the two groups initially given 5-MTHF or FA. Thus, the advantage of the natural folate 5-MTHF is its ability to reliably replete body stores in folate insuficient women within a few days. The new serum status, while decreasing slightly on the fourth day can be then maintained by a typical prenatal dose of FA.

For the purpose of data analysis, a target serum level was selected based on several studies of women of childbearing age with baseline plasma total folate between about 18 nM and 21 nM (measured by microbiological assay). In these earlier studies subjects were administered FA and followed for 12 or 24 weeks. In the 24 week study, by 12 weeks those given 0.4 mg/d of FA reached a plateau of total plasma folate of about 53 to 55 nM^17^. The other two studies reported plasma levels of 44 nM (dose = 0.375 mg/d) and 51 nM (dose = 0.4 mg/d) after 12 weeks^15,16^. Based on these studies and since it now appears that consumption of 0.4 mg/d FA for several months can lower the rate of NTD to among the lowest yet observed^12^, a plasma/serum concentration of total folate of 50 nM was selected to compare the effects of 5-MTHF with FA.

In the current study only after the third and last dose of 5-MTHF given once per day (group A) was the 50 nM target level for total serum folate reached by all subjects. Moreover after switching to 0.4 mg/d FA, the average serum total folate decreased to 53.8 nM, but about one third of subjects continued to be below target for the next two weeks. On the other hand, all of those in group B (except one subject after 24 hrs) administered five doses every 12 hours were continuously above 50 nM after the first dose. During the two subsequent weeks while taking 0.8 mg/d of 5-MTHF, all except one subject were maintained above 50 nM total serum folate. These findings suggest that five doses of 7.5 mg/d of 5-MTHF given every 12 hours can provide this level almost immediately, which can then be maintained with a lower dose prenatal vitamin. With this regimen a total of 37.5 mg of 5-MTHF is administered, and earlier studies on urinary excretion^20^ suggest that the retained folate would be about 26 mg (57 μmols). This is consistent with the range of the total body stores of folate reported for humans^21,22^, and with the ability of the follow-on prenatal dose to maintain the new status.

Although FA eventually produces the equivalent serum total folate level on average as the same dose of 5-MTHF (group C vs A), a pronounced lag of at least four days was observed. This is likely due to both a higher urinary excretion of FA than 5-MTHF^20^, and its slow conversion by dihydrofolate reductase into active folate forms^23^. This results in exposure to high serum concentration of unmetabolized FA after each dose, and lower serum 5-MTHF for many hours after each dose than if administered 5-MTHF.

Several earlier studies indicate that post conceptional folate can reduce birth defects in both an animal model and in humans. Mouse embryos nullizygous for folate binding protein-1 (Folbp1^−/−^) die in utero showing high percentages of neural tube, craniofacial, and other defects. Such mice can be partially rescued by supplementing the dams with 5-methyl-tetrahydrofolate at high dose^24^. Supplementation during embryonic days seven to nine (during neural fold elevation and tube closure) is necessary to produce this result. Extending provision of folate to embryonic day one through nine, while decreasing the number of resorptions, did not further improve protection from defects. Interestingly, blood samples taken from Folbp1^−/−^ mice 24 hours after a dose did not show an increase in plasma total folate concentration^25^. The authors suggested that “…embryos are able to harvest and use sufficient folate cofactors for their survival and development during the peak plasma folate…” Although a role for Folbp1 has yet to be implicated in humans, these results none the less demonstrate that folate supplied during the narrow period of neural tube fusion can be effective.

In the seminal work of Berry *et al*., women in two regions of China were asked to consume 0.4 mg/d of FA^12^. The northern region had the highest rate of NTD among those who took no folate (4.8 NTD per 1000 registered pregnancies). Among the northern group the rate of NTD dropped to 1.0 per 1000 for those consuming the FA starting before their last menstrual period. However as seen in table 3 of this article, those who started FA during the first trimester, but sometime after their last menstrual period, also had a lower rate of NTD (1.6 per 1000) than unsupplemented women^12^. Further, the outcomes from 17,300 pregnancies in the Hungarian Case Control Surveillance of Congenital Abnormalities were analyzed according to the timing of beginning of FA administration (3 to 6 mg per day). Women who started FA only during the critical period for formation of each abnormality were significantly protected from neural tube and especially orofacial clefts and cardiovascular defects^2^. Thus, late administration of folic acid, though not in an optimal regimen, has a beneficial effect.

Many women do not recognize they are pregnant until after the neural tube closes. Still, the average length of the luteal phase is ~13 days (from the urine luteinizing hormone peak)^26,27^. Thus, about seven to eight days remain before initiation of fusion for those that become soon aware of a missed menses. Many women also use home pregnancy test strips based on human chorionic gonadotropin, and the most sensitive of these can indicate implantation five days earlier. A continuous high-dose folic acid rescue strategy has been proposed in early pregnancy^18^. The results of the current study show that a much more limited administration of the naturally occurring 5-MTHF can quickly replete body stores. It should also be considered that other folate dependent defects, such as of the heart, lip, and palate develop later than closure of the neural tube (Fig. 1).

Clearly, the best protection that can be afforded by folate would be that which increases stores prior to conception. Even in countries with FA fortification a meaningful percentage of women do not have adequate blood folate status to fully reduce risk for defects to the lowest observed rate. Moreover, rapid folate repletion will not be an option for those who do not learn of their pregnancy until after the last folate sensitive event in development. Thus, every effort still must be made to elevate folate in women of child bearing age, especially those who are at increased risk. When possible, rapid folate repletion will be more effective for increasing blood levels within the limited time than administering a typical prenatal vitamin alone. The effectiveness of post-conceptional rapid repletion with 5-MTHF can only be firmly established by comparison with the standard of care administration only of 0.4 mg/d folic acid in a randomized clinical trial. In this regard, the results of the two regimens of 5-MTHF examined provide a guide to the minimum dose of this folate needed for such a study to quickly alleviate insufficiency. In addition, a prospective observational study of plasma folate in women of child bearing age who intend to conceive in the near future and the incidence of birth defects in the resulting offspring would help to establish an optimal level.

## Methods

### Study design and patients

Women of child bearing age, but who had no intention of becoming pregnant within the study period, were recruited from the greater Mobile, Alabama area. Patient informed written consent was obtained, and all procedures were approved by the University of South Alabama IRB. Supplement administration and blood sampling were performed in accordance with relevant guidelines. Potential subjects were initially interviewed to determine if they were regular consumers of vitamin supplements, fortified breakfast cereals, or energy bars. Only those with insignificant intake from these products (about half of initial responders) were screened for baseline folate levels. Those with serum total folate less than 25 nM (the lowest quintile), as measured by the Immulyte 2000 immunoassay system (used only for initial screening), were included. Exclusion criteria were malabsorption conditions, such as Celiac or Crohn’s disease, taking drugs that might interfere with folate uptake or metabolism (such as methotrexate or hydrochlorothiazide), history of cancer, thyroid problems, epilepsy, or having a relative who had already participated. The number of subjects entered into the study groups was based on earlier results comparing the pharmacokinetics of single 5 mg doses of folic acid and 5-MTHF^19^. Subjects were sequentially entered into the three arms of the study. Subjects served as their own control by comparing changes in folate levels between the screening sample and the first study sample taken about one to two weeks later immediately prior to the first test dose.

### Procedures

Subjects were asked to fast and consume no liquids other than water for at least eight hours prior to each visit. Subjects were administered three regimens of folate. Group A (20 subjects) was given 7.5 mg [6S]-5-MTHF calcium salt (16.3 μmols), (Deplin) orally every 24 hrs (three doses total), followed by 0.4 mg/d FA (0.91 μmols/d) for two weeks; group B (21 subjects): 7.5 mg [6S]-5-MTHF every 12 hrs (five doses total), followed by 0.8 mg/d [6S]-5-MTHF calcium salt (1.74 μmols/d) (Solgar) for two weeks; and group C (20 subjects): 7.5 mg USP folic acid (17.0 μmols from Sigma) every 24 hrs (three doses total), followed by 0.4 mg/d FA for two weeks. Cyanocobalamin was administered orally once a day for the first three days of each group, (0.5 mg/day for groups A and C, and 1.0 mg/day for group B). During the morning of the first five days (just prior to any dose) blood was drawn into serum separator tubes, mixed by inverting, let stand at room temperature in the dark for 30 min, and then centrifuged at 1200 × g for 10 min. The resulting serum was transferred into microfuge tubes (in 300 and 500 microliter aliquots), frozen quickly on dry ice, and transferred to a −80 °C freezer. The subjects in groups B and C, and half of those in group A returned on days 12 and 19 for further blood sampling just prior to that morning’s folate dose. Compliance during the two week lower maintenance dose period was assessed by counting remaining tablets.

Serum 5-MTHF was determined by reversed-phase HPLC with detection of its intrinsic fluorescence, and total folate by microbiological assay using the American Tissue Type Culture Collection 7469 strain of Lactobacillus rhamnosus and 5-MTHF as calibrator^28^. Many samples were assayed in duplicate to assure repeatability. Screening samples initially examined by the Immulyte 2000 assay for the purpose of determining eligibility were also measured using the HPLC and microbiological methods.

### Statistical analysis

ANOVA and the t-test were performed using Statistica v10 on square root transformed values which were determined to be normal by the Shapiro-Wilks test. The hypothesis to be tested was that the means of serum total folate or serum 5-MTHF on the four days following a dose would be the same for administration of 5-MTHF in comparison to FA (groups A and C). This was examined by repeated measures general linear ANOVA with a single between-subjects factor (type of folate). The suitability of ANOVA was examined by Levene’s test for homogeneity. Multiple comparisons were corrected by the Bonferroni-Holm method. All P-values are double-sided.

### Data availability

The authors declare that all data supporting the findings of this study are available within the article or from the corresponding author upon reasonable request.
